# A Prolonged Case of Mucocutaneous Leishmaniasis in a Central American Patient

**DOI:** 10.7759/cureus.115266

**Published:** 2026-08-26

**Authors:** Mario M Diaz Moreno, Graciamaria Alvarenga Somoza, Casto D Ramirez Dominguez

**Affiliations:** 1 Internal Medicine, Hospital Nacional San Rafael, Santa Tecla, SLV; 2 Medical School, Universidad Dr. José Matías Delgado, Antiguo Cuscatlán, SLV

**Keywords:** amphotericin b, biopsy, leishmania, mucocutaneous leishmaniasis, travel medicine

## Abstract

Mucocutaneous leishmaniasis (MCL) is a parasitic infection transmitted through sand fly bites. This disease affects people who are living in or traveling to endemic regions around the world. Cases of MCL remain underreported due to its clinical presentation and potential misdiagnosis, with cases in older adults rarely described in the literature. We present a case of an elderly Salvadoran farmer who developed MCL involving the nasal septum following travel abroad and a subsequent ulcerative lesion in the right lower limb. We discuss his diagnosis, treatment, histopathology, and outcome.

## Introduction

Leishmaniasis is a group of diseases associated with infection by parasitic protozoa of the genus *Leishmania*, which are transmitted through the bite of infected phlebotomine sandflies. Cutaneous leishmaniasis (CL) is the most frequent clinical presentation of leishmaniasis, typically characterized by a localized skin lesion at the site of inoculation. Mucosal leishmaniasis (ML) is primarily associated with tissue destruction involving the nasal, oral, and pharyngeal mucosa. Visceral leishmaniasis (VL) is a potentially fatal clinical manifestation caused by hematogenous dissemination of *Leishmania* parasites to other internal organs if left untreated [1]. Texas is the only U.S. state requiring leishmaniasis reporting (since 2007) and recorded a median of six cases annually (range 0-13) from 2008 to 2017. Of these, 41 (59%) had no travel history outside the U.S. Leishmaniasis is not subject to federal reporting requirements, so incidence elsewhere within the U.S. remains speculative [2], and passive surveillance likely underestimates the actual burden. Supporting this, a 2021-2022 molecular reference laboratory study identified 94 cases across 36 states, 75.6% attributed to *Viannia* subgenus species [3]. Systemic treatment for American CL is primarily aimed at preventing mucocutaneous progression, which develops in 2-10% of patients infected with *L. (V.) braziliensis* and, less frequently, *L. (V.) panamensis*, *L. (V.) guyanensis*, or *L. (L.) amazonensis* [4].

## Case presentation

A 65-year-old male farmer from El Salvador with no significant previous medical history was referred from a healthcare center to our institution for specialized evaluation of a 15-year history of progressive cutaneous lesions involving the right lower limb. The lesion initially appeared as an erythematous papule on the distal right thigh and gradually evolved into a 30 × 8 cm ulcer extending to the middle third of the right leg, associated with pain during the previous six months, as well as honey-colored discharge and minimal bleeding for three months. In addition, the patient reported a six-year history of an ulcerative lesion involving the inferior inner border of the right nasal cavity, previously treated at a primary care level as a common ulcer with wound care alone, without improvement, which progressively caused destruction of the nasal septum and distortion of the nasal anatomy, accompanied by pruritus, warmth, and erythema. The patient denied any history of prior trauma to the affected areas, as well as fever, weight loss, night sweats, malaise, or other systemic symptoms. Notably, the patient had traveled to Belize in 2011, where he observed multiple individuals with similar skin lesions.

Physical examination revealed a painless ulcerative nasal lesion with septal destruction, hematic crusts, necrotic areas, serosanguineous discharge, and overlying erythema (Figure 1). Examination of the right lower limb demonstrated a non-tender large ulcer with well-defined borders, bleeding areas, serohematic discharge, and multiple satellite lesions involving the knee and posterior ankle region, while the left lower limb was unremarkable (Figure 2).

**Figure 1:**
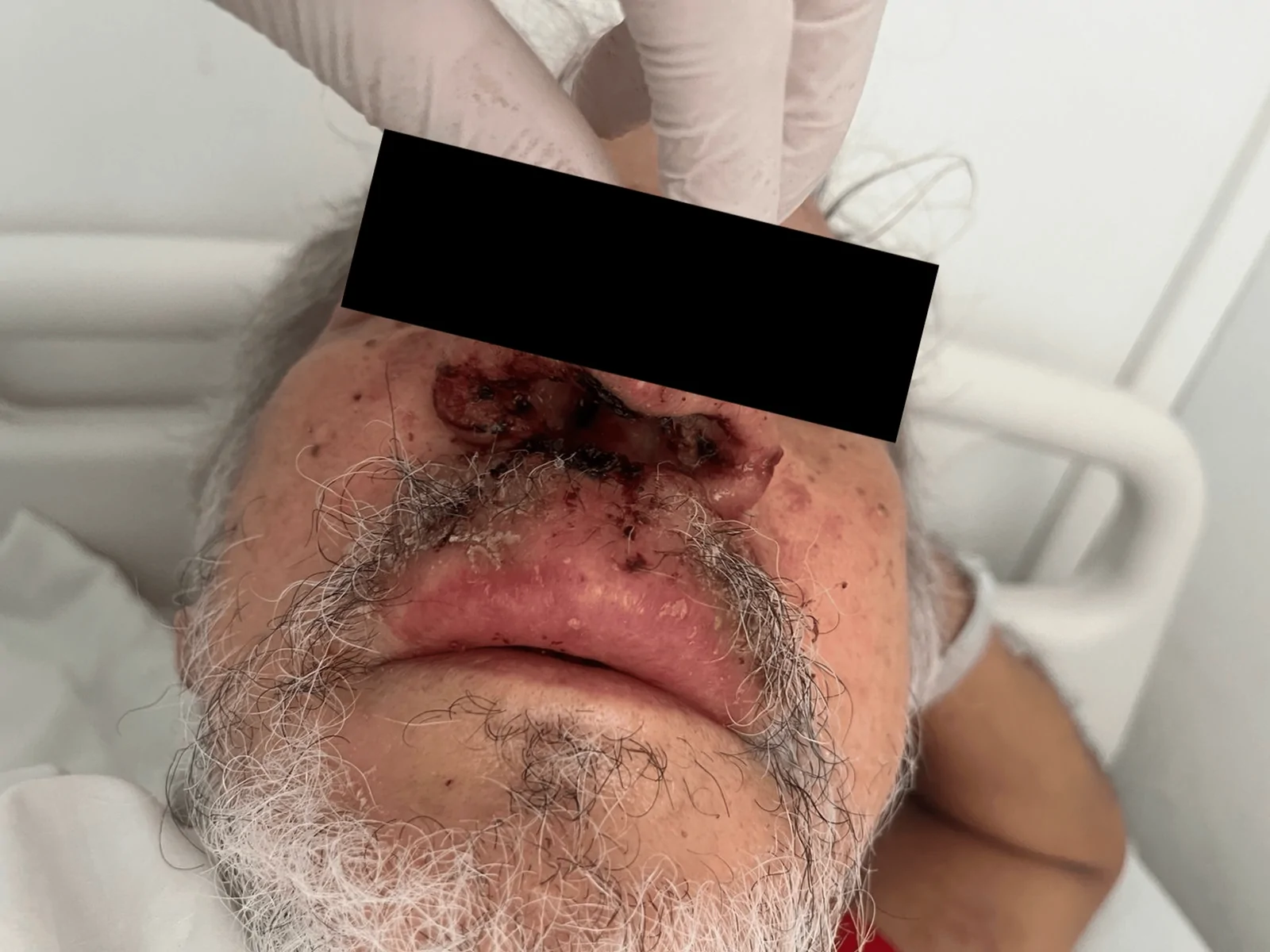
Mucocutaneous leishmaniasis. Ulcerative nasal lesion with septal destruction, hematic crusts, necrotic areas, and serosanguineous discharge.

**Figure 2:**
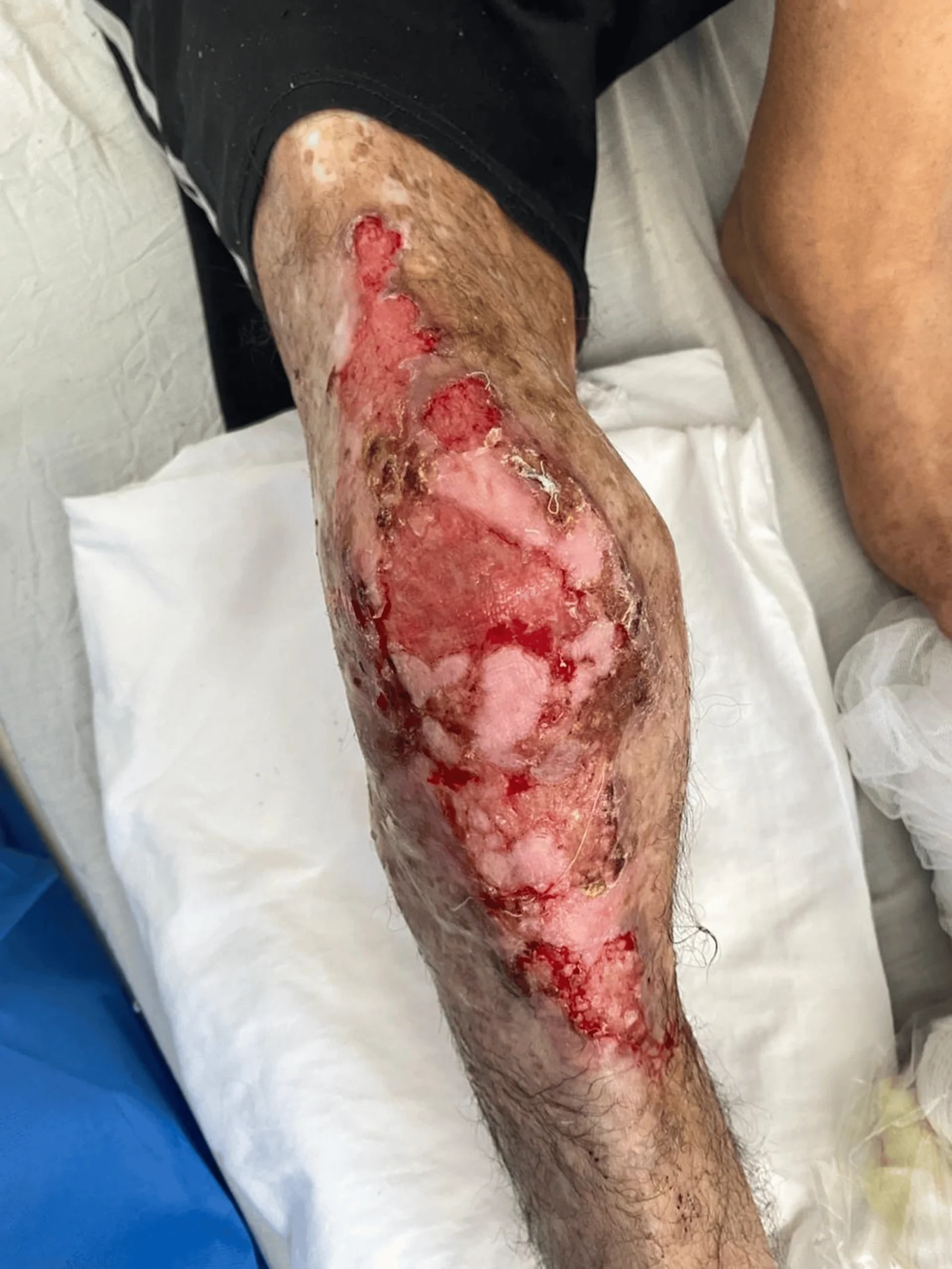
Extensive ulcerative lesion involving the distal thigh and leg of the right lower limb. Marked by exposed granulation tissue, erythematous inflammatory changes, areas of bleeding, serohematic discharge, and multiple satellite lesions.

Further evaluation by Otorhinolaryngology included a biopsy of the nasal lesion. Histopathological analysis revealed extensive inflammatory infiltrates in the superficial dermis consisting of lymphocytes, plasma cells, and macrophages with intracytoplasmic structures compatible with *Leishmania* amastigotes. A smear was taken from the lesion, which was positive (Figure 3). Clinicopathological correlation led to a MCL diagnosis. Subsequently, the case was reported to the department of epidemiology for medication request.

**Figure 3:**
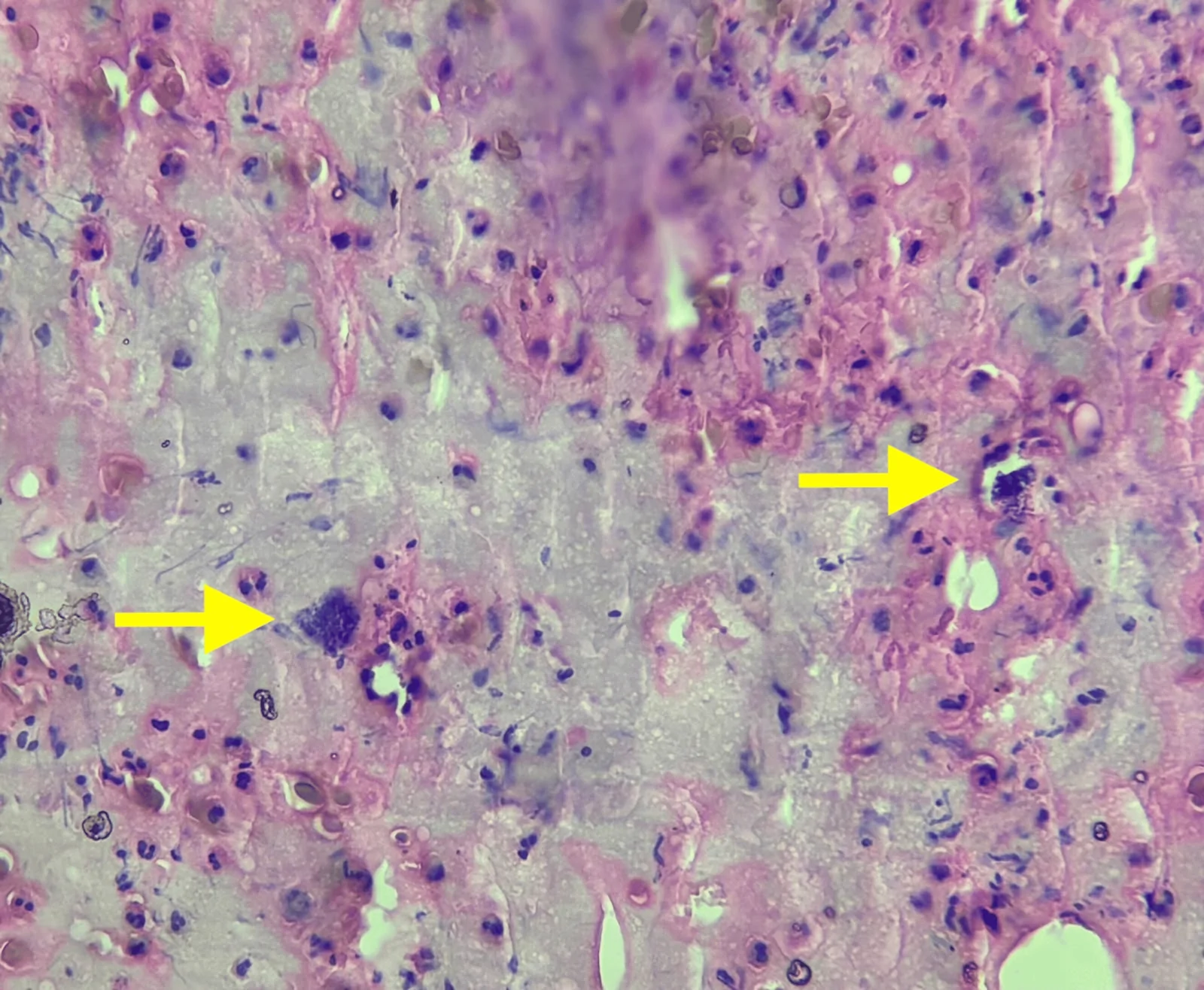
Histopathology of the nasal lesion. Photomicrograph (20x) shows area of ​​necrosis with the presence of two histiocytes with intracytoplasmic amastigotes (yellow arrows).

Culture of the right lower-extremity ulcer was positive for *Staphylococcus aureus*. The wound proved refractory to treatment with broad-spectrum topical antiseptics and systemic ceftriaxone (2 g daily), showing no signs of healing. Systemic therapy was selected following interdisciplinary consultation with the national infectious disease authority, taking into account the risk-benefit profile and the fact that it was the only regimen available within the national healthcare system at the time; initial treatment consisted of liposomal amphotericin B (150 mg) diluted in 250 mL of 5% dextrose administered intravenously over two hours once daily for 10 days.

During hospitalization, the patient responded favorably to liposomal amphotericin B, showing significant clinical improvement. However, he requested discharge against medical advice, so it was decided to continue his management on an outpatient basis with multidisciplinary follow-up, which will include daily wound care and ulcer cleaning at a local health unit.

## Discussion

As a vector-borne infection, leishmaniasis arises from protozoan parasites within the *Leishmania* genus, which reach human hosts through bites from infected Phlebotomus or Lutzomyia sandflies. Currently, leishmaniasis remains endemic across more than 98 countries, accounting for the highest prevalence in Asia, the Americas, and Mediterranean countries, with approximately two million new cases every year [5].

The disease presents in three major clinical forms: CL, MCL, and VL. CL is characterized by cutaneous ulcers, on occasion with satellite lesions or nodular lymphangitis; MCL affects the mucosa along with the underlying connective tissues and cartilage; and VL compromises internal organs, in particular the liver, spleen, and bone marrow. Both VL and MCL can be fatal, although mortality is less frequently observed in MCL [6].

The World Health Organization (WHO) classifies leishmaniasis as a major neglected tropical disease (NTD), considering its impact on public health worldwide. Several risk factors are associated with disease transmission, including poor socioeconomic conditions, malnutrition, migration patterns, poor sanitation, climate change, and restricted access to healthcare services [7].

The epidemiology of leishmaniasis is strongly influenced by climatic conditions. Climate warming may enhance the development of *Leishmania* parasites within the gut of the sand fly vector, consequently accelerating the transition of the vector to an infectious state. Furthermore, humid and rainy conditions favor the formation of suitable microhabitats for vector growth and expansion [7].

According to the Pan American Health Organization (PAHO), leishmaniasis remains endemic across more than a dozen countries in Latin America, where geographic, socioeconomic, and political factors strongly influence transmission patterns [8]. In 2024, the incidence within the region was estimated at 12.76 cases per 100,000 population. Region-specific incidence rates were highest in Suriname, Panama, Honduras, and Guatemala. Among notified cases of CL and ML, approximately 83.7% were laboratory-confirmed, whereas 12.1% were diagnosed based on clinical criteria and an epidemiological link. The remaining 4.2% did not specify the diagnostic criteria used [9].

In 2025, PAHO reported between 16 and 230 CL cases annually in El Salvador from 2018 to 2023, with women accounting for 63% of the cases [9]. In 1986, Ramirez O published a case series of autochthonous CL in El Salvador. As the only prior published case series from this country, it described similarly characterized CL as sporadic disease rather than an epidemic [10]. Belize, on the other hand, first reported CL to PAHO in 2024 with a total of 52 cases, which shows a surveillance gap rather than the true absence of the disease [9]. According to PAHO (2024), the *Leishmania* species associated with CL are *Leishmania infantum* in El Salvador, and *Leishmania braziliensis* and *Leishmania mexicana* in Belize [11].

CL commonly presents as ulcerative skin lesions that may be accompanied by satellite lesions and/or nodular lymphangitis. Due to its diverse clinical presentation, CL represents a significant diagnostic challenge, frequently overlooked and underreported. In some cases, the disease progresses beyond the skin to MCL, which involves the mucosa and underlying connective tissues, including cartilage structures, in combination with CL disease [6]. The incubation period of MCL is one to three months but may occur many years after the initial cutaneous ulcer has healed [12]. VL, also known as kala-azar, represents a potentially life-threatening condition marked by intermittent fever, splenomegaly, hepatomegaly, peripheral lymphadenopathy, and severe anemia. Further manifestations may include unintentional weight loss and hemorrhagic complications, both of which are considered indicators of poor prognosis [13].

Multiple diagnostic methods can be used for the identification of *Leishmania* species. Over the past two decades, PCR molecular techniques have become increasingly common given their high diagnostic accuracy [14]. For CL, the conventional diagnostic approach remains the microscopic identification of amastigotes in Giemsa-stained smears collected from lesional tissue. However, morphological examination alone cannot differentiate among *Leishmania* species. Species-level identification may be achieved through PCR or isoenzyme analysis of cultured parasites, although the latter is labor-intensive, time-consuming, and dependent on parasite isolation in culture.

Despite the availability of advanced diagnostic techniques, their implementation is often limited in resource-constrained settings. In such environments, the visual features of CL make artificial intelligence-powered image analysis and telemedicine promising, adaptable, and financially sustainable alternatives for diagnosis and clinical support [15]. In El Salvador, where molecular diagnostics such as PCR and isoenzyme analysis are not routinely accessible, species-level identification relies predominantly on clinicopathological correlation alongside clinical and geographical context.

The therapeutic management of leishmaniasis is determined by multiple factors, including the clinical considerations, the parasite species involved, efficacy, potential side effects, cost, and the patient's capacity to adhere to the prescribed therapy. First-line therapies for most forms of leishmaniasis consist of pentavalent antimonials, amphotericin B, pentamidine isethionate, and miltefosine. More recently, evidence has supported the use of liposomal amphotericin B for VL and miltefosine for CL due to their improved clinical efficacy and safety profiles [16].

For CL, systemic treatment has traditionally consisted of meglumine antimoniate or sodium stibogluconate, both pentavalent antimonial compounds (Sb5+), regardless of lesion number. Nevertheless, in 2010, the WHO experts encouraged the use of local treatment options for uncomplicated CL in order to minimize systemic toxicity while increasing access to treatment [17].

## Conclusions

This case report shows the importance of considering MCL as a differential diagnosis in patients exhibiting chronic ulcerative skin lesions and extensive mucosal involvement, especially if the person has a history of residence in or travel to endemic regions. The lengthy clinical course and tissue damage emphasize the challenges and consequences associated with delayed recognition of the disease. Histopathological examination remains a valuable diagnostic tool, especially in resource-limited settings where access to advanced molecular techniques may be limited. Furthermore, this case underscores the need to increase awareness of the diverse clinical manifestations of leishmaniasis among healthcare professionals to facilitate diagnosis and prompt treatment, especially in endemic areas where the disease continues to represent an ongoing public health concern.

## References

[1] Aronson NE, Musa AM, Satoskar AR (2026). Leishmaniasis. N Engl J Med.

[2] McIlwee BE, Weis SE, Hosler GA (2018). Incidence of endemic human cutaneous leishmaniasis in the United States. JAMA Dermatol.

[3] Truong TT, Crawford K, Wang-McGuire I (2023). Windows into leishmaniasis epidemiology in the United States: September 2021 through August 2022 [PREPRINT]. medRxiv.

[4] Blaser M, Cohen J, Holland S (2025). Mandell, Douglas, and Bennett's Principles and Practice of Infectious Diseases. 10th Edition.

[5] Madusanka RK, Silva H, Karunaweera ND (2022). Treatment of cutaneous leishmaniasis and insights into species- specific responses: a narrative review. Infect Dis Ther.

[6] de Vries HJ, Schallig HD (2022). Cutaneous leishmaniasis: a 2022 updated narrative review into diagnosis and management developments. Am J Clin Dermatol.

[7] Tapias Rivera J, Martínez-Vega R, Quintero-García WL, Torres-Martínez DS, Monroy-Díaz AL, Sánchez-Corrales L, Gutiérrez-Torres JD (2025). Climatic, socioeconomic, and migratory factors on the epidemiological dynamics of cutaneous leishmaniasis in Colombia, 2007-2021. PLoS Negl Trop Dis.

[8] Olivera MJ, Porras-Villamil JF, Fuentes MV (2025). Spatiotemporal epidemiology of cutaneous, mucosal, and visceral leishmaniasis in Colombia, 1980-2025: insights from a 45-year national analysis. Acta Trop.

[9] Pan American Health Organization (2026). Leishmaniasis: Epidemiological report on the Region of the Americas. No. 14, December 2025. https://iris.paho.org/handle/10665.2/69654.

[10] Ramírez O (1986). Autochthonous cutaneous leishmaniasis in El Salvador. Report of 5 cases [Article in French]. Med Cutan Ibero Lat Am.

[11] Pan American Health Organization (2024). Manual of Procedures for Leishmaniasis Surveillance and Control in the Region of the Americas. Second Edition. https://www.paho.org/en/documents/manual-procedures-leishmaniasis-surveillance-and-control-region-americas-second-ed.

[12] Piscopo TV, Mallia Azzopardi C (2007). Leishmaniasis. Postgrad Med J.

[13] Eddaikra N, Benikhlef R, Sereno D (2025). A century of epidemiological advances in cutaneous and visceral leishmaniasis in Algeria. J Parasitol Res.

[14] Blaizot R, Pasquier G, Kone AK, Duvignaud A, Demar M (2024). Cutaneous leishmaniasis in sub-Saharan Africa: a systematic review of Leishmania species, vectors and reservoirs. Parasit Vectors.

[15] Kim J, Zieneldien T, Ma S, Cohen BA (2025). Cutaneous leishmaniasis in the context of global travel, migration, refugee populations, and humanitarian crises. Clin Pract.

[16] Pan American Health Organization (2022). Guideline for the Treatment of Leishmaniasis in the Americas. Second Edition.

[17] Khattak FA, Khan TA, Hussain M (2022). Analysis of associated risk factors among recurrent cutaneous leishmaniasis patients: a cross-sectional study in Khyber Pakhtunkhwa, Pakistan. J Infect Public Health.

